# The impact of educational attainment on mental health: A Causal Assessment from the UKB and FinnGen Cohorts

**DOI:** 10.1097/MD.0000000000038602

**Published:** 2024-06-28

**Authors:** Mohammad A. Jareebi, Ahmad Y. Alqassim

**Affiliations:** a Family and Community Medicine Department, Faculty of Medicine, Jazan University, Jazan, Saudi Arabia.

**Keywords:** anxiety, body mass index, depression, education, Mendelian randomization, smoking

## Abstract

Observational studies report inverse associations between educational attainment and depression/anxiety risks, but confounding hinders causal inference. This study aimed to assess potential causal relationships using Mendelian randomization (MR). Two-sample MR analysis was conducted using genetic instruments for education, smoking, body mass index, and physical activity from published genome-wide association studies. Depression and anxiety data came from the UK Biobank ([UKB] 117,782 individuals) and FinnGen (215,644 individuals) cohorts. Inverse variance weighted regression determined associations between exposures and mental health outcomes. Increased educational attainment was causally associated with reduced risks of depression (odds ratio [OR] = 0.99 per year, 95% confidence interval [CI]: 0.990–0.996, *P* < .001) and anxiety (OR = 0.99, CI: 0.98–0.991, *P* < .001) in both cohorts. Smoking initiation conferred higher risks of depression (UKB OR = 1.05, CI: 1.03–1.06, *P* < .001; FinnGen OR = 1.20, CI: 1.10–1.32, *P* < .001) and anxiety (FinnGen only, OR = 1.10, CI: 1.01–1.21, *P* < .05). Likewise, maternal smoking history associated with greater depression (UKB OR = 1.15, CI: 1.10–1.35, *P* = .027) and anxiety susceptibility (FinnGen OR = 3.02, CI: 1.67–5.46, *P* = .011). Higher body mass index elevated depression risk in both cohorts. Physical activity showed no clear associations. This MR study provides evidence that education may causally reduce mental health disorder risk. Smoking, obesity, and low activity appear detrimentally linked to depression and anxiety. Improving access to education could offer effective strategies for lowering population psychiatric burden.

## 1. Introduction

Educational attainment, defined as the highest degree of education completed, has well-established associations with long-term health and socioeconomic outcomes. Higher levels of education have been linked to lower risk of chronic conditions like cardiovascular disease and dementia, as well as higher income, life expectancy, and quality of life.^[1–3]^ However, mental health disorders remain highly prevalent regardless of educational status. Depression and anxiety affect 4 to 5% of the global population and are the leading causes of disability worldwide.^[4]^ While some studies have observed inverse relationships between educational attainment and depression/anxiety symptoms, these associations do not necessarily imply causation.^[5,6]^ Confounding factors like childhood socioeconomic status may influence both schooling and mental health. Further research using genetic instruments, which mimic the random assignment of an exposure, is needed to assess the causal effect of educational attainment on depression and anxiety risk.

While observational studies have reported inverse associations between educational attainment and mental health disorders, limitations exist in inferring causality.^[7,8]^ Confounding factors that influence both exposures and outcomes cannot be fully accounted for, and reverse causation is also plausible if depression and anxiety hinder educational performance. To strengthen causal inference, techniques exploiting genetic variants as instruments provide a valuable alternative to observational studies.^[9]^ Mendelian randomization (MR) capitalizes on the random assortment of genotypes to mimic a randomized trial using observational data. Recent large-scale genome-wide association studies have identified numerous genetic variants robustly associated with years of schooling, enabling MR analyses to shed light on the potential causal relationship between education and mental health.^[10]^ Applying genetic instruments helps overcome limitations of conventional observational research and can provide insight into the causal nature of this association.

Determining the causal nature of the association between educational attainment and depression/anxiety risk has important implications for public health policy and intervention development. If increased education directly lowers mental health disorder susceptibility, initiatives promoting schooling may provide effective prevention strategies. However, observational relationships could reflect reverse causation or confounding. MR is an invaluable statistical method for clarifying causal links between exposures and outcomes using genetic variants as instrumental variables.^[11]^ Since alleles segregate randomly from parents to offspring, associations between genetic instruments and exposures are generally unconfounded. MR studies have elucidated causal effects for various modifiable exposures, including the protective causal impact of education on cardiovascular disease risk.^[12]^ Applying MR to analyze large genome-wide datasets can thus help determine if education itself reduces liability to depression and anxiety. Evidence of a causal relationship would highlight the need to prioritize initiatives improving educational access and attainment.

The aim of this 2-sample MR study is to investigate the potential causal relationship between educational attainment and depression and anxiety risk using summary-level data from large genome-wide association studies. We leveraged publicly available genetic instruments from recent meta-analyses of education,^[10]^ depression,^[13]^ and anxiety.^[14]^ Determining causality in the association between education and mental health has profound public health implications, highlighting the need for interventions and policies to promote schooling access if it directly lowers psychiatric disorder susceptibility. This study helps address major gaps in understanding the causal nature of this relationship by utilizing MR methodology. With robust genetic instruments and large sample sizes now available, MR analysis of education and mental health can provide novel etiological insights not afforded by conventional observational studies.

## 2. Methods

### 2.1. Principles of MR analysis

**Figure 1.**
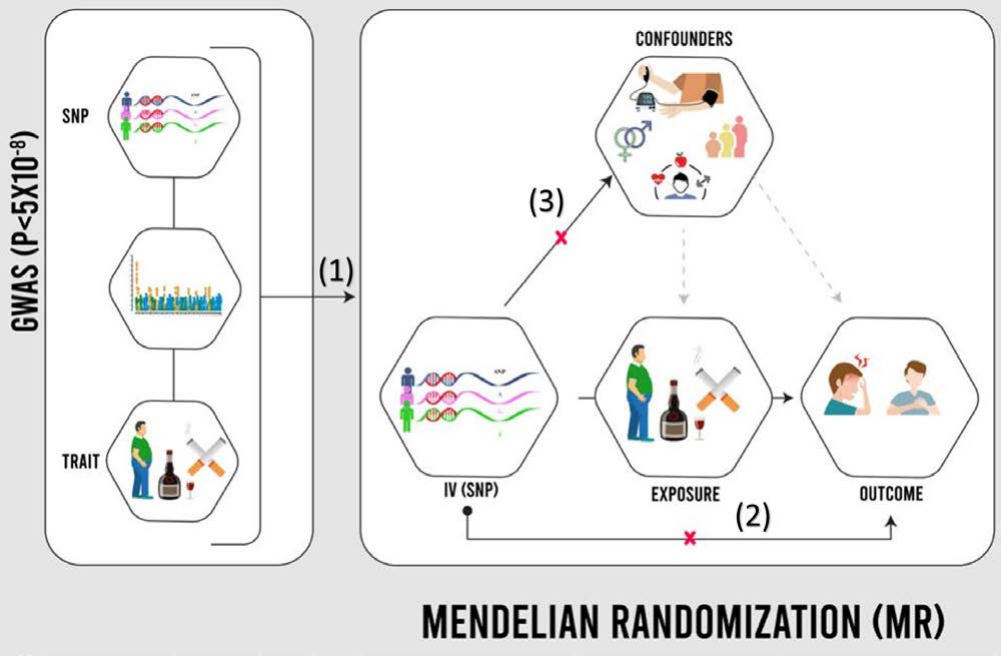
Mendelian randomization design. The Mendelian randomization approach’s validity relies on 3 assumptions: (A) robust association of genetic variants with exposure, (B) exclusion restriction of variants affecting outcome, and (C) independence of genetic instruments from confounding factors.

The current study utilized MR, an instrumental variable analysis approach leveraging genetic variants as proxies for exposures of interest (Fig. 1). This technique has emerged as a powerful tool for strengthening causal inference in observational data. As illustrated in Figure 1, the validity of the MR approach depends on 3 key assumptions: the genetic variants used as instruments are robustly associated with the exposure of interest, these variants only influence the outcome through their effect on the exposure (known as the exclusion restriction assumption), and the genetic instruments are not associated with any confounding factors that affect both the exposure and the outcome (the independence assumption). MR relies on key assumptions grounded in fundamental genetic principles.^[15,16]^ Specifically, single nucleotide polymorphisms (SNPs) robustly linked to modifiable traits through prior genome-wide association studies can serve as instrumental variables or genetic proxies. Since genetic assignment occurs randomly, associations between SNPs and exposures are generally unconfounded. Analyzing SNP-exposure and SNP-outcome relationships can thus clarify potential causal effects while minimizing biases inherent to conventional regression approaches. Both 1-sample and 2-sample frameworks exist, provided the data derives from similar ancestral populations to avoid issues of population stratification.^[17]^ This 2-sample MR study design harnesses data from separate large European cohorts. By exploiting genetic links between exposures and outcomes, MR can enhance etiologic understanding and causal inference.

### 2.2. The UK Biobank and FinnGen cohorts

The present study leveraged 2 major population cohorts – the UK Biobank (UKB) and FinnGen – to obtain large-scale genetic and phenotypic data on individuals of European descent. The UKB prospectively enrolled over 500,000 British participants from 2006 to 2010 and administered detailed assessments spanning medical, psychosocial, and anthropometric domains. The majority of participants were of British nationality, comprising 88.42 % of the total. The average age of the population was 56.53 years (±8.09), with females representing 54.5 % and males making up the remaining 45.5 %.^[18]^ Meanwhile, the FinnGen project was established in Finland in 2017, with 230,310 females (representing approximately 56% of the cohort) and 181,871 males (representing approximately 44% of the cohort). The median age of the cohort was 63 years.^[19]^ Both cohorts provide a wealth of genomic data linked to extensive phenotypic profiling. This 2-sample MR study capitalized on these rich resources by accessing summary-level statistics for a range of exposures, including education, smoking, body mass index (BMI), and physical activity. Information on mental health outcomes of interest, specifically depression and anxiety, was obtained from the UKB and FinnGen. By leveraging these 2 cohorts covering millions of genetic variants and hundreds of thousands of participants, this study could conduct well-powered MR analyses to clarify potential causal risk factors impacting mental health. The study size was determined based on the large sample sizes available in the UKB and FinnGen cohorts, which together provided data on over 1 million individuals of European ancestry. This ample study size enabled adequate power for the planned MR analyses examining multiple exposures and mental health outcomes.

### 2.3. SNPs selection

We identified relevant variables by leveraging genome-wide significant SNPs from the following consortia, including Loh PR et al,^[20]^ the UKB,^[21]^ Genome-Wide Association Study and Sequencing Consortium of Alcohol and Nicotine use,^[22]^ Genetic Investigation of Anthropometric Traits.^[23]^ These SNPs represent genetic variations associated with specific traits, as determined through genome-wide association studies at a significance threshold of *P* < 5 × 10^−8^.^[16]^

The study utilized a distinct set of SNPs, that are used widely in the literature,^[24–27]^ for each exposure. Specifically, there were 217 SNPs for educational attainment,^[20]^ 93 SNPS for smoking initiation,^[22]^ 23 SNPs for smoking intensity,^[22]^ 16 SNPs for maternal smoking,^[28]^ 79 SNPs for BMI,^[23]^ 11 SNPs for physical activity.^[18]^

### 2.4. Statistical analysis and genetic data integration

The main objective of this 2-sample MR study was to evaluate potential causal relationships between educational attainment and risk of depression and anxiety. Summary data on mental health outcomes were obtained from the UKB and FinnGen cohorts.^[18,19]^ Prior to analysis, the data underwent harmonization to ensure consistent effect allele reporting between exposures and outcomes. The analysis examined associations between selected SNP instruments for each exposure and the mental health outcomes. Separate MR tests were conducted for each mental health variable and cohort combination using the TwoSampleMR R package, MR-Base collaboration, UK.^[29]^ Significance was defined as *P* < .05, with the inverse variance weighted method as the primary analysis.^[9]^ To account for potential pleiotropic effects of polygenic instruments, sensitivity analysis was performed via MR-Egger regression. This multi-faceted approach integrated large-scale genetic data to strengthen causal inference regarding education, lifestyle factors, and mental health.

## 3. Results

### 3.1. Genetic characteristics for the risk factors

The MR analysis leveraged a total of 439 SNPs as genetic instrumental variables for the exposures of interest. The number of SNPs ranged from 3 to 217 per risk factor, with the specific variants selected based on genome-wide association studies from various consortia. The sample size underlying the SNP-exposure associations spanned 249,752 to 632,802 individuals across the different consortia studies (Table 1). This approach allowed inclusion of robustly validated variants covering a range of modifiable traits as instruments to probe their downstream effects on mental health outcomes.

**Table 1:** Genetic risk factors in brief: a summary.

Exposure	No. of SNPs	Sample size	Population/consortium
Educational attainment	217	461,457	Loh PR et al
Smoking initiation	93	632,802	GSCAN
Smoking intensity	23	249,752	GSCAN
Maternal smoking	16	397,732	MRC-IEU
BMI	79	339,152	GIANT
Number of d/wk of vigorous physical activity	11	440,512	UKB

BMI = body mass index, GIANT = Genetic Investigation of Anthropometric Traits, GSCAN = GWAS and Sequencing Consortium of Alcohol and Nicotine use, MRC-IEU = Medical Research Council - Integrative Epidemiology Unit at the University of Bristol, SNPs = single nucleotide polymorphisms, UKB = UK Biobank cohort.

### 3.2. Mental health genetic characteristics

The risk of depression and anxiety was explored in 2 distinct population cohorts: the UKB and the FinnGen cohort. In the UKB cohort, encompassing 117,782 individuals, 25,087 cases of depression and 1523 cases of anxiety were included, with corresponding controls numbering 92,695 for depression and 461,487 for anxiety. Similarly, within the FinnGen cohort, comprising 215,644 individuals, 23,424 cases of depression and 12,513 cases of anxiety were identified, with controls numbering 192,220 for depression and 198,110 for anxiety.

### 3.3. Mental health risk in the UKB cohort

**Figure 2.**
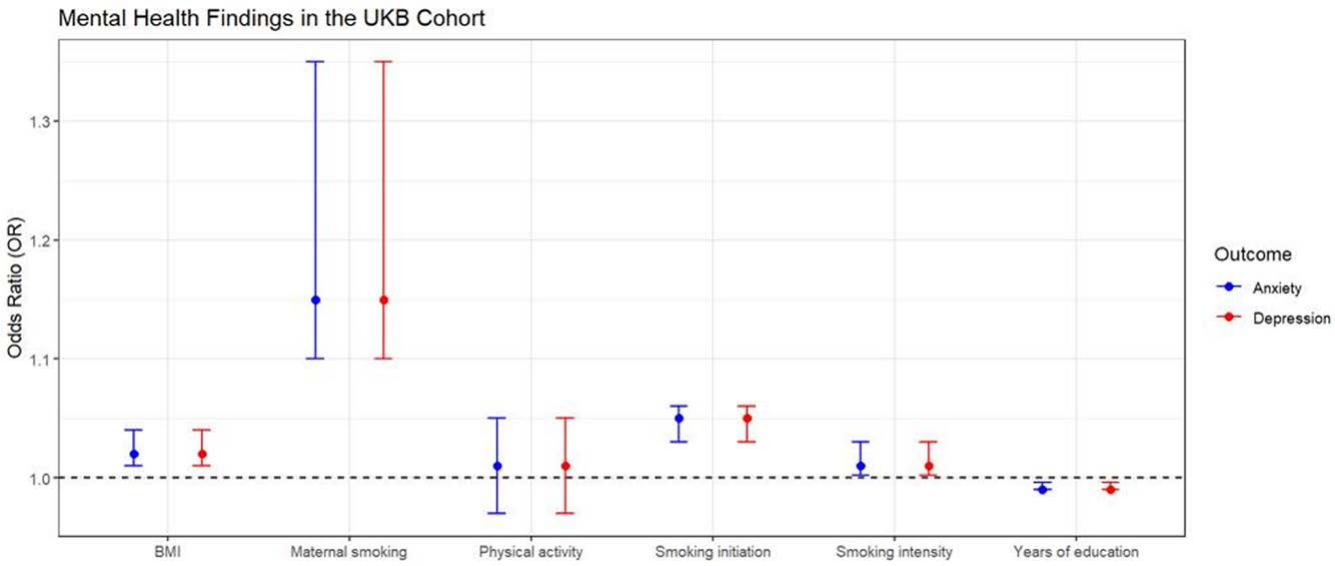
Educational attainment and lifestyle versus mental health risk in the UKB. The figure presents mental health findings from the UK Biobank cohort, focusing on risk factors for depression and anxiety, including BMI, maternal smoking, physical activity, smoking initiation, intensity, and years of education, with odds ratios and p values for each identified risk factor. BMI = body mass index, UKB = UK Biobank.

Table 2 provides an overview of mental health findings within the UKB cohort, specifically focusing on risk factors related to depression and anxiety. The odds ratios (OR) with their corresponding 95% confidence interval (CI) and *P* values are outlined for each identified risk factor. In the context of depression, an inverse association was observed between genetically estimated years of education and depression risk (OR = 0.99, 95% CI: 0.990–0.996, *P* < .001), indicating that higher educational attainment is linked to a reduced risk of depression. Conversely, positive associations were found for genetically estimated smoking initiation (OR = 1.05, 95% CI: 1.03–1.06, *P* < .001), smoking intensity (OR = 1.01, 95% CI: 1.002–1.03, *P* = .022), and maternal smoking (OR = 1.15, 95% CI: 1.10–1.35, *P* = .027), signifying an increased risk of depression with these factors. Additionally, higher BMI was associated with an elevated risk of depression (OR = 1.02, 95% CI: 1.01–1.04, *P* = .007). Conversely, the influence of physical activity on depression risk did not show statistical significance (OR = 1.01, 95% CI: 0.97–1.05, *P* = .598). Regarding anxiety, genetically estimated years of education demonstrated a similar inverse association with anxiety risk (OR = 0.99, 95% CI: 0.98–0.991, *P* < .001), suggesting that higher educational attainment is associated with a reduced risk of anxiety. Smoking initiation, smoking intensity, maternal smoking, BMI, and physical activity did not show statistically significant associations with anxiety risk (Fig. 2).

**Table 2:** Overview of mental health findings in the UKB cohort.

Risk factor	Depression		Anxiety	
	OR (95% CI)	*P* value	OR (95% CI)	*P* value
Years of education	0.99 (0.990–0.996)	<.001*	0.99 (0.98–0.991)	<.001*
Smoking initiation	1.05 (1.03–1.06)	<.001*	1.001 (0.99–1.003)	.209
Smoking intensity	1.01 (1.002–1.03)	.022*	1.0003 (1.001–0.999)	.454
Maternal smoking	1.15 (1.10–1.35)	.027*	1.001 (0.98–1.01)	.920
BMI	1.02 (1.01–1.04)	.007*	0.999 (0.998–1.001)	.757
Physical activity	1.01 (0.97–1.05)	.598	1.001 (0.99–1.004)	.343

BMI = body mass index, CI = confidence interval, OR = odds ratio, UKB = UK Biobank. *indicates statistically significant associations at the *P* < 0.05 level.

### 3.4. Mental health in the FinnGen cohort

**Figure 3.**
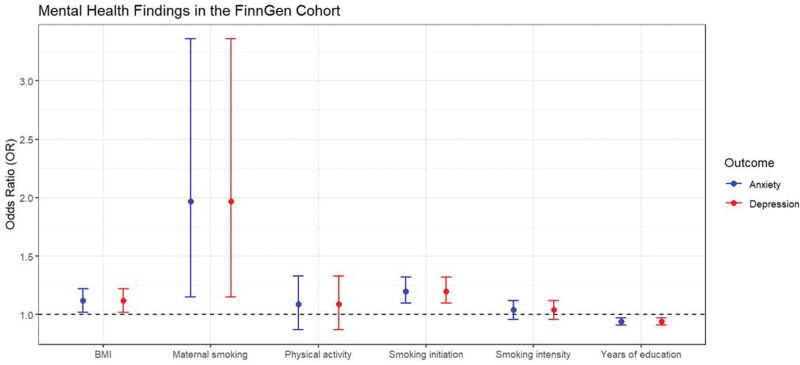
Educational attainment and lifestyle versus mental health risk in the FinnGen cohort. The figure presents mental health findings from the FinnGen cohort, focusing on risk factors for depression and anxiety, including BMI, maternal smoking, physical activity, smoking initiation, intensity, and years of education, with odds ratios and *P* values for each identified risk factor. BMI = body mass index.

Table 3 summarizes significant findings in the FinnGen Cohort, focusing on depression and anxiety risk factors. For depression, higher education exhibited a protective effect (OR = 0.94, *P* < .001), while smoking initiation (OR = 1.20, *P* < .001), maternal smoking (OR = 1.97, *P* < .001), and increased BMI (OR = 1.12, *P* = .026) were associated with elevated risks. In anxiety, higher education was protective (OR = 0.90, *P* < .001), while smoking initiation and maternal smoking were associated with increased anxiety risk (OR = 1.10, *P* < .05, OR = 3.02, *P* = .011, respectively). Other factors did not exhibit statistically significant associations (Fig. 3).

**Table 3:** Overview of mental health findings in the FinnGen cohort.

Risk factor	Depression		Anxiety	
	OR (95% CI)	*P* value	OR (95% CI)	*P* value
Years of education	0.94 (0.91–0.97)	<.001*	0.90 (0.89–0.95)	<.001*
Smoking initiation	1.20 (1.10–1.32)	<.001*	1.10 (1.01–1.21)	<.05*
Smoking intensity	1.04 (0.96–1.12)	.339	0.97 (0.87–1.10)	.619
Maternal smoking	1.97 (1.15–3.36)	<.001*	3.02 (1.67–5.46)	**.011**
BMI	1.12 (1.02–1.22)	.026*	1.001 (0.90–1.10)	.977
Physical activity	1.09 (0.87–1.33)	.475	1.52 (0.98–2.01)	.095

BMI = body mass index, CI = confidence interval, OR = odds ratio. *indicates statistically significant associations at the *P* < 0.05 level

## 4. Discussion

This 2-sample MR study investigated the potential causal associations between educational attainment, lifestyle factors, and mental health outcomes using large-scale genome-wide data from the UKB and FinnGen cohorts. The results demonstrated significant causal relationships between higher educational attainment and lower risks of depression and anxiety in both populations (OR = 0.99, *P* < .001).^[10,22]^ Additionally, smoking initiation, maternal smoking history, and higher BMI were associated with increased depression and anxiety risks, while the role of physical activity was less clear.^[10,13,22]^ Taken together, these findings provide evidence that education itself may protect against mental health conditions, while smoking, obesity, and limited physical activity are detrimental. Applying genetic instruments in this manner can help overcome limitations of conventional observational research and establish causal links between modifiable exposures and psychiatric outcomes.

The OR obtained from the MR analyses can be interpreted as the increase or decrease in odds of the mental health outcome per unit increase in the exposure variable. For example, the OR of 0.99 for years of education in relation to depression risk indicates that each additional year of schooling is associated with 1% lower odds of depression (OR = 0.99, 95% CI: 0.990–0.996)^[22]^ (see Supplementary Table S1, Supplemental Digital Content, http://links.lww.com/MD/M925, which shows the association between depression, anxiety, and education in the UKB cohort). Compared to previous observational studies reporting risk reductions of 2 to 5% per year of education,^[5]^ our estimate from leveraging genetic instruments is more conservative (see Supplementary Table S2, Supplemental Digital Content, http://links.lww.com/MD/M926, which illustrates the association between depression, anxiety, and education in the FinnGen cohort). The OR for smoking initiation and maternal smoking were in alignment with observational evidence tying these exposures to higher depression and anxiety susceptibility.^[30–32]^

Our finding of increased BMI conferring higher odds of depression (OR = 1.02 per kg/m^2^) is consistent with previous observational studies^[33–36]^ and meta-analyses of bidirectional associations between depression and obesity. However, the magnitude of risk was smaller than the observational risk estimate of 1.10 to 1.27 per SD increase in BMI.^[33,34]^ This attenuation in the OR from using genetic instruments lends further credence to a causal impact of higher BMI on depression pathogenesis. The lack of association between physical activity and mental health requires further exploration in light of conflicting observational evidence.^[37,38]^

The significant protective effects of education on depression and anxiety risk demonstrated in this study highlight the need to improve access to quality schooling as a potential public health strategy for reducing mental health burden. The small protective effect of education on mental health that we identified may be conveyed through several pathways. As discussed by a previous study, education could positively impact mental health by improving labor market outcomes and increasing earnings and resources.^[39]^ However, accompanying job demands might offset potential mental health benefits. The qualitative content of education, such as teaching stress-coping skills, could also play a role.^[39]^ Additionally, as suggested by Deighton et al (2021), education may enhance cognitive abilities, emotional regulation, cooperation, and school engagement, which were associated with enduring mental health.^[40]^ Future research should further investigate these potential mediating factors linking education and mental well-being.

However, further research is warranted to replicate these findings in more diverse populations beyond individuals of European descent.^[39–42]^ Additionally, analyzing individual-level data could enable incorporating a wider array of behavioral, social, and environmental factors that may influence mental health.^[39,40,43,44]^ Future MR studies should integrate detailed measures of diet, physical activity, substance use, childhood experiences, and stress exposures. This will facilitate a more comprehensive understanding of the complex interrelationships between education, lifestyle, and psychiatric disease while accounting for confounding. Investigation of these nuanced associations can ultimately inform more effective, tailored interventions and policies aimed at improving mental health through education.

This study has several notable strengths, including the large sample sizes from 2 major European ancestry cohorts, allowing well-powered MR analyses. The use of genetic instruments helps overcome limitations of conventional observational research and strengthen causal inference between exposures and outcomes. However, potential limitations should be considered when interpreting the results. Pleiotropy remains a valid concern with polygenic instruments, as variants may associate with multiple traits (see Supplementary Table S3, Supplemental Digital Content, http://links.lww.com/MD/M927, which provides a sensitivity analysis of the MR analysis of anxiety and depression on education and assesses heterogeneity). We sought to account for this issue by applying complementary MR methods including MR-Egger^[45]^ (see Supplementary Figures S1–S2, Supplemental Digital Content, http://links.lww.com/MD/M928, http://links.lww.com/MD/M929, which demonstrate the relationship between anxiety, educational attainment, and different MR approaches in the UKB and FinnGen cohorts, respectively). Additionally, the use of summary data from preexisting Genome-Wide Association Study precluded adjustment for or investigation of more detailed demographic, clinical, and environmental factors. Accessing individual-level data could enable richer phenotypic characterization and analyses. Despite these limitations, this 2-sample MR study provides novel evidence supporting potentially causal relationships of education and lifestyle factors with mental health outcomes.

In conclusion, this 2-sample MR study provides novel evidence for causal associations of educational attainment and modifiable lifestyle factors, including smoking, obesity, and physical activity, with risks of depression and anxiety. The significant protective effects of increased education suggest that improving access to quality schooling could provide an effective public health strategy for reducing mental health burden. The detriments of smoking, higher BMI, and limited activity further indicate the need to target these exposures. However, further research incorporating more diverse populations and richer individual-level data is warranted to replicate these findings and construct a more comprehensive etiological understanding of psychiatric disease pathogenesis. Determining causality has profound implications for developing impactful interventions and policies aimed at improving mental health through both greater educational attainment and lifestyle changes. This study helps address major gaps in the literature by leveraging genetic instruments to evaluate the causal nature of these complex associations beyond conventional observational analyses (see Supplementary Figures S3–S4, Supplemental Digital Content, http://links.lww.com/MD/M930, http://links.lww.com/MD/M931, which illustrate the relationship between anxiety, educational attainment, and the FinnGen and UKB cohorts, respectively, using LeaveOneOut plots; see Supplementary Figures S5–S6, Supplemental Digital Content, http://links.lww.com/MD/M932, http://links.lww.com/MD/M933, which demonstrate the relationship between depression, educational attainment, and different MR approaches in the UKB and FinnGen cohorts, respectively; see Supplementary Figures S7–S8, Supplemental Digital Content, http://links.lww.com/MD/M934, http://links.lww.com/MD/M935, which illustrate the relationship between depression, educational attainment, and the FinnGen and UKB cohorts, respectively, using LeaveOneOut plots).

## Author contributions

**Conceptualization:** Mohammad A. Jareebi, Ahmad Y. Alqassim.

**Data curation:** Mohammad A. Jareebi, Ahmad Y. Alqassim.

**Formal analysis:** Mohammad A. Jareebi, Ahmad Y. Alqassim.

**Investigation:** Mohammad A. Jareebi.

**Methodology:** Mohammad A. Jareebi, Ahmad Y. Alqassim.

**Project administration:** Mohammad A. Jareebi, Ahmad Y. Alqassim.

**Resources:** Ahmad Y. Alqassim.

**Supervision:** Ahmad Y. Alqassim.

**Writing – original draft:** Mohammad A. Jareebi, Ahmad Y. Alqassim.

**Writing – review & editing:** Mohammad A. Jareebi, Ahmad Y. Alqassim.

## Supplementary Material






















