# Supplementary material for: The impact of educational attainment on mental health: A Causal Assessment from the UKB and FinnGen Cohorts
Source: Medicine (Baltimore). 2024 Jun 28;103(26):e38602. doi: 10.1097/MD.0000000000038602 (PMC11466082; doi:10.1097/MD.0000000000038602)
Supplement: Supplementary file 1 [file medi-103-e38602-s001.docx]

| **Table S1.** Association between depression, anxiety, and education in the UKB cohort | | | | |
| --- | --- | --- | --- | --- |
|  | **Depression** | | **Anxiety** | |
| **Risk factor** | **OR (95% CI)** | **P value** | **OR (95% CI)** | **P value** |
| Years of education | 0.98 (0.988 - 0.989) | **0.018** | 0.998 (0.996 - 0.998) | **0.017** |

**Variable IDs:**

Educational attainment (years of education) id: ebi-a-GCST90029013

**UKB:**

Depression id: ukb-d-F5_DEPRESSIO

Anxiety id: ukb-b-11311
