# Supplementary material for: The impact of educational attainment on mental health: A Causal Assessment from the UKB and FinnGen Cohorts
Source: Medicine (Baltimore). 2024 Jun 28;103(26):e38602. doi: 10.1097/MD.0000000000038602 (PMC11466082; doi:10.1097/MD.0000000000038602)
Supplement: Supplementary file 3 [file medi-103-e38602-s003.docx]

| **Table S3.** Sensitivity analysis of the MR analysis of anxiety and depression on education: heterogeneity assessment | |
| --- | --- |
| **Method** | **P value** |
| Depression (UKB) |  |
| *MR Egger* | 0.3856 |
| *Inverse variance weighted* | 0.4012 |
| Anxiety (UKB) |  |
| *MR Egger* | 0.988 |
| *Inverse variance weighted* | 0.9832 |
| Depression (FinnGen) |  |
| *MR Egger* | 0.000002141 |
| *Inverse variance weighted* | 0.000002226 |
| Anxiety (FinnGen) |  |
| *MR Egger* | 0.007006 |
| *Inverse variance weighted* | 0.007745 |

In the sensitivity analysis for depression in the UKB cohort, both MR Egger and inverse variance weighted methods yielded p-values greater than 0.05, suggesting no significant heterogeneity. Similarly, for anxiety in the UKB cohort, both methods yielded p-values well above 0.05, indicating consistency in the results. However, in the FinnGen cohort, the sensitivity analysis for depression and anxiety using both MR Egger and inverse variance weighted methods showed significantly low p-values, suggesting potential heterogeneity. This implies that while the association between educational attainment and mental health outcomes remains consistent in the UKB cohort, there may be some heterogeneity present in the FinnGen cohort.
