# Supplementary material for: The impact of educational attainment on mental health: A Causal Assessment from the UKB and FinnGen Cohorts
Source: Medicine (Baltimore). 2024 Jun 28;103(26):e38602. doi: 10.1097/MD.0000000000038602 (PMC11466082; doi:10.1097/MD.0000000000038602)
Supplement: Supplementary file 11 [file medi-103-e38602-s011.docx]

Figure S8. Depression and educational attainment in the UKB cohort LeaveOneOut plot
